# Association Between Differences in Estimated GFR by Creatinine Vs. Cystatin C and Stroke Outcomes: Results From the Third China National Stroke Registry

**DOI:** 10.1002/cns.70930

**Published:** 2026-05-18

**Authors:** Qi Huang, Yuesong Pan, Hongyi Yan, Xia Meng, Yin Zhang, Mengxing Wang, Anxin Wang, Yilun Zhou, Yongjun Wang

**Affiliations:** ^1^ Department of Nephrology, Beijing Tiantan Hospital Capital Medical University Beijing China; ^2^ Department of Epidemiology and Health Statistics School of Public Health, Capital Medical University Beijing China; ^3^ Department of Neurology, Beijing Tiantan Hospital Capital Medical University Beijing China; ^4^ China National Clinical Research Center for Neurological Diseases Beijing China; ^5^ Center of Stroke Beijing Institute for Brain Disorders Beijing China; ^6^ Beijing Key Laboratory of Translational Medicine for Cerebrovascular Disease Beijing China

**Keywords:** creatinine, cystatin C, glomerular filtration rate, prognosis, stroke

## Abstract

**Background:**

The effect of the difference between cystatin C‐ and creatinine‐based estimated glomerular filtration rates (eGFRdiff) on stroke outcomes is unclear. This study investigated the association between eGFRdiff and stroke prognosis.

**Methods:**

Participants with ischaemic stroke or transient ischaemic attack were recruited from the Third China National Stroke Registry, a multicenter, prospective cohort. eGFRdiff was calculated using the absolute difference (eGFRabdiff defined as cystatin C‐based (eGFRcys) minus creatinine‐based (eGFRcr)) and the ratio (eGFRrediff) between eGFRcys and eGFRcr estimates. eGFRabdiff (< −15, −15–15, ≥ 15 mL/min/1.73 m^2^) and eGFRrediff (< 0.6, ≥ 0.6) were analyzed. Time‐updated eGFRdiff (ΔeGFRdiff) was defined as the change from baseline to 1‐year blood re‐examination and tertiled. Outcomes included 5‐year all‐cause mortality, stroke disability, and stroke recurrence.

**Results:**

Among 10,293 participants, 35.2% had an eGFRabdiff < −15 mL/min/1.73 m^2^, and 3.5% had an eGFRrediff < 0.6. Compared with midrange eGFRabdiff, participants with eGFRabdiff < −15 mL/min/1.73 m^2^ had an odds ratio (OR) of 1.19 (95% confidence interval (CI), 1.05–1.35) for disability and a hazard ratio (HR) of 1.28 (95% CI, 1.12–1.46) for mortality. eGFRrediff < 0.6 was associated with a higher risk for disability (OR, 1.66; 95% CI, 1.25–2.19) and mortality (HR, 1.49; 95% CI, 1.17–1.90). Participants with the largest ΔeGFRdiff declines had higher mortality (OR, 1.44; 95% CI, 1.14–1.82) and borderline disability risk significance (HR, 1.25; 95% CI, 0.96–1.61).

**Conclusions:**

Significant and widening eGFRdiff were independently associated with increased risks of post‐stroke disability and mortality, highlighting the importance of monitoring eGFRcys and eGFRcr in stroke management.

## Graphical abstract

Discrepancies between cystatin C‐based and creatinine‐based eGFR (eGFRdiff) have been linked to adverse outcomes in conditions such as kidney disease and cardiovascular disorders. However, the impact of eGFRdiff on stroke outcomes remains unexplored, necessitating this study. This study is the first to demonstrate that significant and widening eGFRdiff is independently associated with increased risks of post‐stroke disability and mortality.

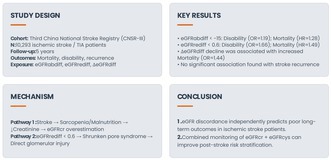

## 1 Introduction

Serum creatinine or cystatin C is commonly used to estimate glomerular filtration rate (eGFR) in medical practice, which is often employed to assess kidney function and predict adverse prognosis in stroke patients. Although eGFRs based on creatinine (eGFRcr) and cystatin C are highly correlated, discrepancies may exist between their values [1, 2]. A study based on data from the UK Biobank found that over 40% of participants had an eGFRcys that was at least 20% lower than their eGFRcr [2], and such differences are more prone to occur in patients with poorer health conditions in clinical settings.

In recent years, large intraindividual differences between eGFRcys and eGFRcr have become increasingly recognized and have continued evidence linking them to a series of adverse events, including frailty, falls, hospitalizations, diabetes‐related multimorbidity risks, peripheral artery disease, cardiovascular events, higher prevalence and incidence rates of stroke, mortality, and renal failure [3, 4, 5, 6, 7, 8, 9, 10, 11, 12, 13, 14]. Moreover, two studies focused on changes in eGFRdiffcys‐cr over time have indicated that participants with a faster decline in eGFRcys had a higher risk of adverse outcomes than those with eGFRcr [3, 9]. The correlation between the differences and adverse prognosis may be related to the decreased predictive ability of eGFRcr in specific populations, such as those with advanced age, extreme muscle mass, and severe chronic illnesses [15, 16].

To our knowledge, there are currently no studies on the association between the difference in eGFRcys and eGFRcr (eGFRdiff) and stroke prognosis. Additionally, due to muscle mass loss in chronic illness, particularly in disability following stroke, eGFR based on serum creatinine may overestimate kidney function. The Third China National Stroke Registry (CNSR‐III) is a cohort study with a 5‐year follow‐up of patients with acute ischaemic stroke or transient ischaemic attack (TIA). Serum creatinine and cystatin C levels were centrally measured at admission and 1 year. Therefore, we analyzed data from CNSR‐III to answer the following two questions: (1) Is baseline eGFRdiffcys‐cr independently associated with 5‐year stroke outcomes? (2) Does time‐updated eGFRdiffcys‐cr associate with stroke outcomes?

## 2 Methods

### 2.1 Study Population

The study population included patients from the CNSR‐III. The CNSR‐III is a national multicenter prospective registry that evaluates the etiology, imaging, and biological markers associated with ischaemic stroke or TIA diagnosis. Patients older than 18 years and within 7 days of symptom onset were enrolled at 201 study sites. The details of the CNSR‐III cohort have been described previously [17]. In the CNSR‐III cohort, 15,166 patients were enrolled between August 2015 and March 2018. In the present study, we included participants with centrally measured serum creatinine and cystatin C levels and 5‐year follow‐up data from CNSR‐III. Written informed consent was obtained from all patients or their legally authorized representatives. The CNSR‐III study was performed in accordance with the Declaration of Helsinki, and was approved by the ethics committee at Beijing Tiantan Hospital (IRB approval number: KY2015‐001–01) and all participating centers. Written informed consent was obtained from the patient or legally authorized representative (primarily spouse, parents, adult children, otherwise indicated).

The baseline data of the included patients were collected within 24 h after admission through face‐to‐face interviews by research coordinators. Medical history included diabetes, hypertension, hyperlipidaemia, and coronary heart disease. The baseline information included age, sex, and body mass index. Stroke severity was assessed using the National Institutes of Health Stroke Scale (NIHSS). The medications used during hospitalization were also recorded, including dehydrants, contrast agents, and angiotensin‐converting enzyme inhibitors/angiotensin receptor blockers. Independent data monitoring was performed by an independent contract research organization using an electronic data capture system throughout the study period. All data were de‐identified prior to the analysis.

Blood samples were collected from all patients within 24 h of admission and 12 months post‐interview. All blood samples were processed and transported via cold chain to the central laboratory at Beijing Tiantan Hospital, where all serum specimens were stored at −80°C until testing.

### 2.2 Major Study Measurements

Serum creatinine levels were measured using an enzymatic method (sarcosine oxidase‐PAP) with a commercial kit (Beckman Coulter, Brea, CA, USA) following the manufacturer's protocol. The Beckman assay was calibrated using the Roche/Hitachi P module Creatinase Plus enzymatic assay (Roche Diagnostics, Basel, Switzerland) and was traceable to an isotope dilution mass spectrometry assay at the National Institute of Standards and Technology. Cystatin C levels were measured using an immunoturbidimetric method (Roche Cobas c501 analyzer with Tina‐quant Cystatin C Gen.2 assay) with an approximate coefficient of variation of 2%.

### 2.3 Independent Variable

The independent variable of interest was eGFRdiffcys‐cr, which included the absolute difference in eGFR (eGFRabdiff), defined as eGFRcys minus eGFRcr, and the relative difference in eGFR (eGFRrediff), defined as eGFRcys/eGFRcr. The eGFRabdiff was further stratified by three categories: (1) < −15 mL/min/1.73 m^2^ (negative eGFRabdiff), (2) –15–15 mL/min/1.73 m^2^ (midrange eGFRabdiff), and (3) ≥ 15 mL/min/1.73 m^2^ (positive eGFRabdiff), which was consistent with published evidence [3, 5, 9]. Additionally, 15 mL/min per 1.73 m2 corresponds to one standard deviation (SD) and approximates the difference in eGFRs between chronic kidney disease categories. While the eGFRrediff was categorized into two groups: < 0.6 and ≥ 0.6, we chose 0.6 as the cutoff value primarily based on previous studies [5, 18] and the pathophysiological basis suggested by the shrinkage syndrome (see discussion section for details). The 2012 Chronic Kidney Disease Epidemiology Collaboration equations were used to calculate eGFRcys, eGFRcr, and eGFRcr‐cys. The updated 2021 equations were not used due to their low accuracy in risk prediction in the Chinese population.

The time‐updated eGFRdiffcys‐cr (Δ eGFRdiff) was defined as 1‐year blood re‐examination eGFRdiffcys‐cr minus baseline eGFRdiffcys‐cr, and then tertiled into three groups from low to high: Tertile 1, Tertile 2, and Tertile 3.

### 2.4 Outcomes

Primary outcomes included all‐cause mortality, stroke disability, and stroke recurrence at 5‐years post‐enrolment. All‐cause mortality was confirmed by death certificates from the attending hospital or the local citizen registry. Poor functional outcome was defined using mRS scores ranging from 3 to 6. Stroke recurrence was defined as a new‐onset focal neurological deficit induced by cerebral ischaemic or haemorrhagic events and was confirmed using computed tomography or magnetic resonance imaging.

### 2.5 Statistical Analyses

The baseline characteristics were compared between the eGFRdiff groups. Continuous variables were expressed as means with standard deviations or medians with interquartile ranges, and categorical variables as numbers with percentages. Continuous variables were compared between groups using the *t*‐test or Mann–Whitney *U* test, and categorical variables were compared using the chi‐square test.

The associations between eGFRdiff or ΔeGFRdiff and all‐cause mortality or stroke recurrence were evaluated using multivariable Cox proportional hazard regression models, with hazard ratios (HRs) and their 95% confidence intervals (CIs) calculated. Multivariable logistic regression was used to determine stroke disability outcomes with odds ratios (ORs) and 95% CIs.

Model 1 was adjusted for age, sex, and baseline eGFRcr, as it is the most common measure of GFR assessed in clinical practice. Model 2 was further adjusted for hypertension, coronary artery disease, diabetes mellitus, hyperlipidaemia, smoking, alcohol consumption, body mass index, high‐density lipoprotein cholesterol, low‐density lipoprotein cholesterol (LDL‐C), triglycerides (TG), baseline NIHSS score, angiotensin‐converting enzyme inhibitors/angiotensin receptor blockers, dehydrant and contrast agent use, and baseline log urine albumin–creatinine ratio.

All data were analyzed using the SAS version 9.4 software (SAS Institute Inc., Cary, NC, USA). A two‐sided significance level of *p* < 0.05 (two‐sided) was set for all analyses.

## 3 Results

### 3.1 Baseline Characteristics

Among the 15,166 patients enrolled in CNSR‐III, we excluded 2563 who did not participate in the biomarker study, 1342 whose blood samples were not sent to the central laboratory, 837 who did not have serum Cr or CysC data at baseline, and 131 who did not have 5‐year follow‐up data. The final analysis included 10,293 patients. The mean (SD) age of the included patients was 62.3 (11.3) years; 7024 (68.2%) were males. At baseline, mean (SD) eGFRcys was 80.6 (21.8) mL/min/1.73 m^2^, and mean (SD) eGFRcr was 89.7 (18.2) mL/min/1.73 m^2^; moreover, the majority of participants (74.4%) had lower eGFRcys values than eGFRcr values, with a mean (SD) of −9.2 (15.5) mL/min/1.73 m^2^. Over one‐third of participants exhibited an eGFRdiffcys‐cr lower than −15 mL/min/1.73 m^2^ (negative eGFRdiffcys‐cr; 3620 participants, 35.2%). Additionally, 6159 participants (59.8%) had midrange eGFRabdiff, and 514 (5.0%) showed positive eGFRabdiff. Among the participants, 9934 (96.5%) had eGFRrediff ≥ 0.6, and 359 (3.5%) had eGFRrediff < 0.6. Participants with negative eGFRabdiff were generally older and had a higher prevalence of smoking, a history of coronary artery disease, and more frequent use of dehydrants. Furthermore, they had the lowest diastolic blood pressure, LDL‐C, and TG levels but the highest uric acid level among the three eGFRabdiff groups (Table 1).

**TABLE 1:** Baseline characteristics of participants from the CNSR‐III trial overall and categorized of eGFRdiffcys‐cr.

Variable	Overall	eGFRabdiffcys‐cr, ml/min/1.73 m^2^				eGFrediff cys/cr		
		Negative < −15 (eGFRcys lower than eGFRcr)	Mid‐range−15 to 15 (eGFRcys similar to eGFRcr)	Positive ≥ 15 (eG FRcys higher than eGFRcr)	*p*	≥ 0.6	< 0.6	*p*
Participants, *n* (%)	10,293	3,620 (35.16)	6,159 (59.84)	514 (4.99)	NA	9,934	395	NA
Age, y	62.33 ± 11.31	64.19 ± 11.33	61.48 ± 11.13	59.50 ± 11.50	< 0.0001	62.12 ± 11.26	68.38 ± 11.20	< 0.0001
Male, *n* (%)	7,027 (68.27)	2,510 (69.34)	4,160 (67.54)	357 (69.46)	0.1544	6,774 (68.19)	253 (70.47)	0.3868
Current smoking, *n* (%)	3,248 (31.56)	1,242 (34.31)	1,871 (30.38)	135 (26.26)	< 0.0001	3,134 (31.55)	114 (31.75)	0.9539
Current alcohol drinking, *n* (%)	1,456 (14.14)	511 (14.12)	878 (14.26)	67 (13.04)	0.7460	1,419 (14.28)	37 (10.31)	0.0370
Hypertension, *n* (%)	6,483 (62.98)	2,305 (63.67)	3,842 (62.38)	336 (65.37)	0.2280	6,238 (62.79)	245 (68.25)	0.0394
SBP, mmHg	150.24 ± 22.23	150.72 ± 22.40	150.09 ± 22.35	148.58 ± 19.20	0.1355	150.30 ± 22.22	148.42 ± 22.34	0.0908
DBP, mmHg	87.41 ± 13.17	86.80 ± 13.10	87.70 ± 13.20	88.18 ± 13.38	0.0007	87.50 ± 13.17	84.81 ± 13.16	< 0.0001
Diabetes mellitus, *n* (%)	2,449 (23.79)	830 (22.93)	1,504 (24.42)	115 (22.37)	0.1829	2,356 (23.72)	93 (25.91)	0.3441
Hyperlipidemia, *n* (%)	857 (8.33)	310 (8.56)	496 (8.05)	51 (9.92)	0.2748	827 (8.32)	30 (8.36)	1.0000
Coronary artery disease, *n* (%)	1,122 (10.90)	449 (12.40)	626 (10.16)	47 (9.14)	0.0012	1,066 (10.73)	56 (15.6)	0.0056
Baseline NIHSS, score	3 (1, 6)	3 (1, 6)	3 (1, 6)	4 (2, 6)	0.0482	3 (1, 6)	4 (2, 6)	0.0023
Body Mass Index, kg/m^2^	24.70 ± 3.32	24.74 ± 3.41	24.66 ± 3.26	24.89 ± 3.51	0.4621	24.70 ± 3.32	24.52 ± 3.50	0.1518
HDL‐C, mmol/L	0.97 ± 0.29	0.96 ± 0.28	0.97 ± 0.28	1.04 ± 0.44	0.0510	0.97 ± 0.29	0.93 ± 0.30	0.0101
LDL‐C, mmol/L	2.45 ± 1.10	2.40 ± 0.96	2.42 ± 0.99	3.12 ± 1.96	< 0.0001	2.46 ± 1.06	2.31 ± 1.06	0.0012
Triglyceride, mmol/L	1.57 ± 0.88	1.46 ± 0.74	1.56 ± 0.84	2.44 ± 1.58	< 0.0001	1.58 ± 0.88	1.52 ± 0.94	0.0977
Serum creatinine, μmol/L	73.44 ± 24.74	69.80 ± 15.78	73.44 ± 26.26	98.93 ± 38.71	< 0.0001	73.34 ± 24.82	75.88 ± 22.32	0.0008
Cystatin C, mg/L	1.00 ± 0.28	1.14 ± 0.23	0.93 ± 0.27	0.80 ± 0.19	< 0.0001	0.98 ± 0.25	1.54 ± 0.36	< 0.0001
Uric acid	302.74 ± 90.70	311.79 ± 91.89	297.40 ± 86.68	301.21 ± 88.92	< 0.0001	300.98 ± ±88.70	350.78 ± 125.06	< 0.0001
Medication								
ACEI, *n* (%)	464 (9.14)	167 (9.14)	275 (9.20)	22 (8.49)	0.9309	451 (9.22)	13 (6.99)	0.3638
ARB, *n* (%)	1,313 (25.86)	466 (25.49)	786 (26.3)	61 (23.55)	0.5641	1,263 (25.83)	50 (26.88)	0.7335
Dehydrant, *n* (%)	519 (5.08)	146 (4.06)	340 (5.57)	33 (6.48)	0.0017	502 (5.09)	17 (4.84)	1.0000
Contrast agent, *n* (%)	218 (2.12)	62 (1.71)	142 (2.31)	14 (2.72)	0.0897	209 (2.10)	9 (2.51)	0.5731
UACR, mg/g	6.87 (2.16–27.44)	7.43 (2.17–30.52)	6.70 (2.16–26.87)	5.95 (2.14–20.69)	0.0743	6.68 (2.13–26.60)	19.37 (4.42–105.48)	< 0.0001
eGFRcys, ml/min/1.73 m^2^	80.60 ± 21.82	66.50 ± 15.12	87.27 ± 20.78	99.88 ± 21.18	< 0.0001	81.92 ± 20.96	44.12 ± 11.10	< 0.0001
eGFRcr, ml/min/1.73 m^2^	89.77 ± 18.27	91.33 ± 14.30	90.25 ± 19.05	72.96 ± 24.43	< 0.0001	89.97 ± 18.22	84.17 ± 18.57	< 0.0001
eGFRdiffcys‐cr, ml/min/1.73 m^2^	−9.17 ± 15.51	−24.83 ± 8.43	−2.98 ± 7.52	26.91 ± 12.26	< 0.0001	−8.06 ± 14.44	−40.06 ± 11.59	< 0.0001

*Note:* Mean values and their corresponding SDs are presented for continuous variables. Categorical variables are presented as numbers and percentages.

Abbreviations: ACEI, angiotensin converting enzyme inhibitors; ARB, angiotensin receptor blockers; eGFR, estimated glomerular filtration rate; HDL‐C, high‐density lipoprotein cholesterol; LDL‐C, low‐density lipoprotein cholesterol; NHISS, National Institute of Health Stroke Scale; UACR, urine albumin creatine ratio.

### 3.2 Baseline eGFRdiff and Stroke Outcomes at 5 Years

**FIGURE 1:**
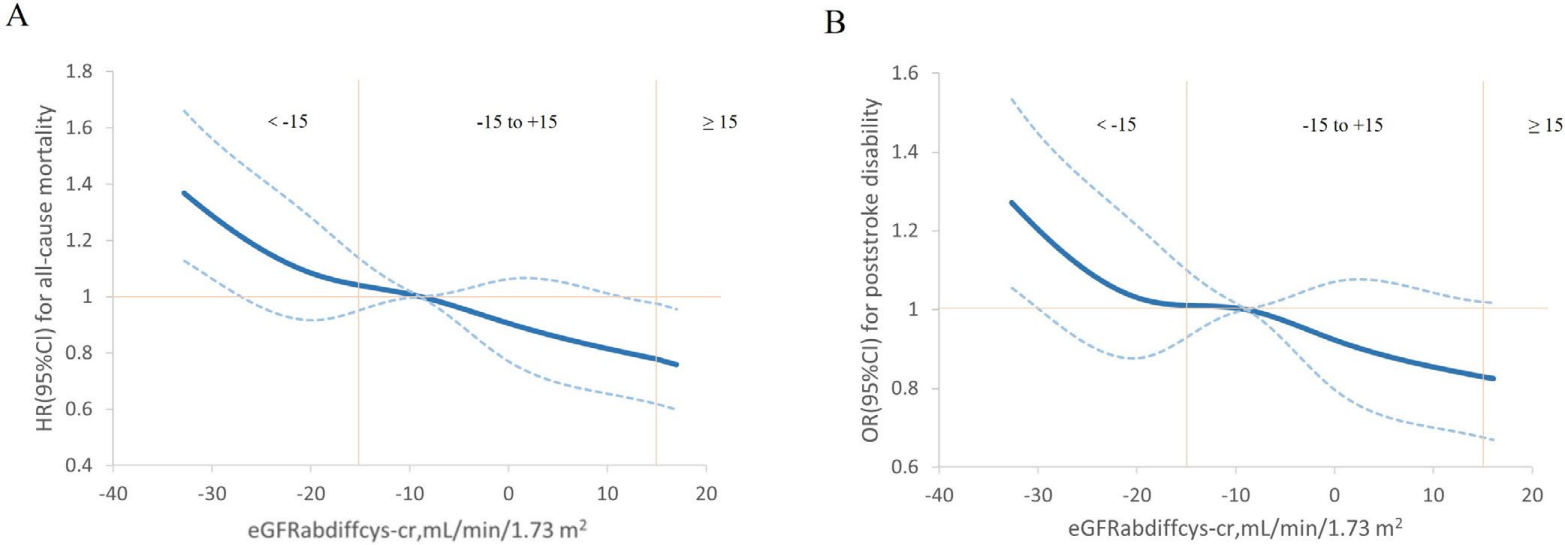
Dose–response relationship between eGFRabdiff and 5‐year all‐cause mortality (A) and stroke disability (B). A restricted cubic spline was used to explore nonlinear associations. The HR was derived using Cox proportional hazard regression, and logistic regression was used to calculate the OR, which adjusted for age, sex, baseline eGFRcr, hypertension, coronary artery disease, diabetes mellitus, hyperlipidaemia, smoking, alcohol consumption, body mass index, high‐density lipoprotein cholesterol, low‐density lipoprotein cholesterol, triglycerides, baseline NIHSS score, use of angiotensin‐converting enzyme inhibitors/angiotensin receptor blockers, dehydrant and contrast agent, and baseline log urine albumin–creatinine ratio.

During the follow‐up of 5 years, post‐stroke disability developed in 1852 participants, and all‐cause mortality occurred in 1017 participants. Compared with participants with a midrange eGFRabdiff, those with a negative eGFRabdiff exhibited an adjusted OR of 1.19 (95% CI, 1.05–1.35) for post‐stroke disability and an HR of 1.28 (1.12–1.46) for all‐cause mortality. Conversely, participants with a positive eGFRabdiff showed an OR of 0.82 (0.59–1.14) and an HR of 0.78 (0.54–1.13). A decrease of 1‐SD in eGFRabdiff correlated with a 14% increased risk of post‐stroke disability and a 20% elevated risk of all‐cause mortality (Tables 3 and 4). As the negative value of eGFRabdiff decreases, the risk of death and post‐stroke disability gradually declines (Figure 1A,B). Compared with participants with an eGFRrediff ≥ 0.6, those with an eGFRrediff < 0.6 exhibited a higher risk, with an OR of 1.66 (95% CI, 1.25–2.19) for stroke disability and a HR of 1.49 (1.17–1.90) for all‐cause mortality. For each 10% reduction in eGFRrediff, the corresponding ORs and HRs were 1.05 (1.02–1.08) and 1.07 (1.04–1.11), respectively (Tables 3 and 4).

The rates of stroke recurrence at 5 years across the groups were 16.22% (*n* = 587), 15.91% (*n* = 980), and 16.34% (*n* = 84), respectively, with no significant differences (Supplemental data Table S1).

### 3.3 ΔeGFRdiff And Stroke Outcomes at 5 Years

This study included 4508 patients (mean age, 61.4 years; 68.5% males) with baseline and 1‐year data on serum Cr and CysC levels. The mean eGFRcys was 82.2 (SD = 21.0) mL/min/1.73 m^2^ at baseline, decreasing to 73.66 (SD = 20.89) at 1 year. Conversely, the mean eGFRcr was 91.1 (SD = 17.4) mL/min/1.73 m^2^ at baseline, increasing to 93.01 (SD = 17.4) at 1 year. The mean ΔeGFRdiff was −10.38 (SD = 16.54) mL/min/1.73 m^2^. Participants with a large negative ΔeGFRdiff were predominantly older, more likely to be female, and exhibited the lowest TG among the three ΔeGFRdiff groups (Table 2). In the adjusted model incorporating time‐updated variables, participants in the first tertile of the ΔeGFRdiff group (the largest negative) exhibited a higher risk of mortality than the midrange ΔeGFRdiff group (OR = 1.44; 95% CI, 1.04–2.00) (Table 3). This group also showed borderline significance for post‐stroke disability (HR = 1.25; 95% CI, 0.96–1.61; *p* = 0.086) in model 2, with a significant difference in model 1 (HR = 1.447; 95% CI, 1.148–1.824; *p* = 0.002) and the unadjusted model (HR = 1.316; 95% CI, 1.057–1.840; *p* = 0.001) (Table 4).

**TABLE 2:** 1‐year time‐updated characteristics of participants from the CNSR‐III trial categorized by overall and ΔeGFRdiffcys‐cr.

Variable	Overall	ΔeGFRdiffcys‐cr, ml/min/1.73 m^2^			
		Tertile 1 (negative change)	Tertile 2 (unchanged)	Tertile 3 (positive change)	*p*
Participants, *n* (%)	4508	1,506	1500	1502	
Age, y	61.42 ± 10.92	61.99 ± 11.18	61.73 ± 10.60	60.53 ± 10.94	0.0014
Male, *n* (%)	3,092 (68.58)	1,005 (66.73)	1,023 (68.2)	1,064 (70.84)	0.0487
Current smoking, *n* (%)	1,475 (32.72)	484 (32.14)	484 (32.27)	507 (33.75)	0.5762
Current alcohol drinking, *n* (%)	679 (15.06)	223 (14.81)	211 (14.07)	245 (16.31)	0.1253
Hypertension, *n* (%)	2,795 (62.00)	907 (60.23)	943 (62.87)	945 (62.92)	0.2203
SBP, mmHg	150.60 ± 22.03	151.68 ± 22.51	150.83 ± 21.88	149.29 ± 21.65	0.0591
DBP, mmHg	87.64 ± 13.02	87.66 ± 13.22	87.38 ± 12.84	87.88 ± 13.11	0.6194
Diabetes mellitus, *n* (%)	1,020 (22.62)	338 (2.44)	332 (22.13)	350 (23.30)	0.7302
Hyperlipidemia, *n* (%)	368 (8.16)	126 (8.37)	101 (6.73)	141 (9.39)	0.0576
Coronary artery disease, *n* (%)	436 (9.67)	149 (9.89)	139 (9.27)	148 (9.85)	0.8092
Baseline NIHSS, score	3 (1–5)	3 (1–5)	3 (1–5)	3 (1–6)	0.0659
Body Mass Index, kg/m^2^	24.80 ± 3.36	24.83 ± 3.29	24.84 ± 3.45	24.75 ± 3.36	0.4753
HDL‐C, mmol/L	0.98 ± 0.28	0.98 ± 0.28	0.97 ± 0.26	0.97 ± 0.30	0.0870
LDL‐C, mmol/L	2.44 ± 1.02	2.42 ± 0.96	2.38 ± 0.90	2.52 ± 1.19	0.1752
Triglyceride, mmol/L	1.56 ± 0.85	1.47 ± 0.70	1.51 ± 0.75	1.72 ± 1.04	< 0.0001
Serum creatinine, μmol/L	72.30 ± 22.75	70.29 ± 21.04	70.62 ± 20.76	75.79 ± 25.68	< 0.0001
Cystatin C, mg/L	0.98 ± 0.25	1.06 ± 0.28	0.97 ± 0.23	0.90 ± 0.21	< 0.0001
Uric acid, μmol/L	303.25 ± 89.56	305.17 ± 88.98	302.98 ± 89.12	301.36 ± 90.75	0.4111
Medication					
ACEI, *n* (%)	219 (9.81)	78 (10.40)	68 (9.14)	73 (9.89)	0.7124
ARB, *n* (%)	517 (23.16)	188 (25.07)	164 (22.04)	165 (22.36)	0.3134
Dehydrant, *n* (%)	179 (4.00)	46 (3.07)	59 (3.96)	74 (4.95)	0.0311
Contrast agent, *n* (%)	71 (1.57)	19 (1.26)	17 (1.13)	35 (2.33)	0.0152
UACR, mg/g	5.89 (1.97–22.21)	6.21 (1.88–21.80)	5.25 (1.80–20.69)	6.33 (2.10–25.00)	0.0525
eGFRcys_base_, ml/min/1.73 m^2^	82.16 ± 21.02	90.05 ± 19.64	82.06 ± 19.93	74.41 ± 20.53	< 0.0001
eGFRcr_base_, ml/min/1.73 m^2^	91.13 ± 17.42	88.83 ± 18.95	92.17 ± 16.31	92.39 ± 16.66	< 0.0001
eGFRabdiff_base_, ml/min/1.73 m^2^	−8.96 ± 15.06	1.21 ± 14.23	−10.10 ± 11.28	−17.98 ± 12.84	< 0.0001
eGFRcys_1‐year_, ml/min/1.73 m^2^	73.66 ± 20.89	70.50 ± 17.38	73.14 ± 19.66	77.30 ± 24.43	< 0.0001
eGFRcr_1‐year_, ml/min/1.73 m^2^	93.01 ± 17.4	96.96 ± 14.02	92.98 ± 15.84	89.09 ± 20.75	< 0.0001
eGFRabdiff_1‐year_, ml/min/1.73 m^2^	−19.35 ± 13.58	−26.45 ± 12.20	−19.83 ± 11.45	−11.79 ± 12.85	< 0.0001
ΔeGFRdiffcys‐cr, ml/min/1.73 m^2^	−10.38 ± 16.54	−27.67 ± 11.54	−10.10 ± 11.28	6.18 ± 10.07	< 0.0001

*Note:* Mean values and their corresponding SDs are presented for continuous variables. Categorical variables are presented as numbers and percentages.

Abbreviations: ACEI, angiotensin converting enzyme inhibitors; ARB, angiotensin receptor blockers; eGFR, estimated glomerular filtration rate; HDL‐C, high‐density lipoprotein cholesterol; LDL‐C, low‐density lipoprotein cholesterol; NHISS, National Institute of Health Stroke Scale; UACR, urine albumin creatine ratio.

**TABLE 3:** Association of baseline and time‐updated eGFRdiffcys‐cr with Stroke disability.

Measure	Unadjusted		Model 1		Model 2	
	OR/HR (95% CI)	*p*	OR/HR (95% CI)	*p*	OR/HR (95% CI)	*p*
*Baseline measures*						
Categorical eGFRdiffcys‐cr, mL/min/1.73 m2						
< −15	1.332 (1.198–1.481)	< 0.0001	1.162 (1.037–1.302)	0.0100	1.190 (1.047–1.352)	0.0077
−15 to 15	1.0 (ref)		1.0 (ref)		1.0 (ref)	
≥ 15	0.838 (0.647–1.084)	0.1790	0.711 (0.536–0.943)	0.0179	0.821 (0.593–1.138)	0.2372
Per 1‐SD decrease	1.199 (1.138–1.262)	< 0.0001	1.146 (1.082–1.215)	< 0.0001	1.145 (1.072–1.225)	< 0.0001
Categorical eGFRcys/eGFRcr						
≥ 0.6	1.0 (ref)		1.0 (ref)		1.0 (ref)	
< 0.6	2.714 (2.150–3.425)	< 0.0001	1.867 (1.454–2.399)	< 0.0001	1.659 (1.254–2.194)	0.0004
Per 10% decrease	1.119 (1.088–1.152)	< 0.0001	1.062 (1.034–1.089)	< 0.0001	1.052 (1.019–1.086)	0.0016
*1‐year time‐updated measures*						
Categorical ΔeGFRdiffcys‐cr, mL/min/1.73 m2						
Tertile 1 (negative change)	1.316 (1.057–1.840)	0.0014	1.447 (1.148–1.824)	0.0017	1.251 (0.969–1.614)	0.0855
Tertile 2 (unchanged)	1.0 (ref)		1.0 (ref)		1.0 (ref)	
Tertile 3 (positive change)	1.164 (0.931–1.456)	0.1831	1.142 (0.904–1.444)	0.2647	1.074 (0.832–1.385)	0.5856

*Note:* Model 1: Analysis adjusted for age, sex, and baseline creatinine‐based eGFR. Model 2: Model 1 plus hypertension, coronary artery disease, diabetes mellitus, hyperlipidemia, smoking, alcohol consumption, body mass index, HDL‐C, LDL‐C, triglyceride, baseline NIHSS score, ACEI/ARB, dehydrant and contrast agent use, and baseline log UACR (data need log transformed).

**TABLE 4:** Association of baseline and time‐updated eGFRdiffcys‐cr with All‐cause mortality.

Measure	Unadjusted		Model 1		Model 2	
	OR/HR (95% CI)	*p*	OR/HR (95% CI)	*p*	OR/HR (95% CI)	*p*
*Baseline measures*						
Categorical eGFRdiffcys‐cr, mL/min/1.73 m2						
< −15	1.453 (1.285–1.642)	< 0.0001	1.292 (1.139–1.466)	< 0.0001	1.280 (1.119–1.464)	0.0003
−15 to 15	1.0 (ref)		1.0 (ref)		1.0 (ref)	
≥ 15	0.819 (0.592–1.134)	0.2290	0.675 (0.483–0.943)	0.0212	0.779 (0.538–1.129)	0.1872
Per 1‐SD decrease	1.259 (1.184–1.338)	< 0.0001	1.230 (1.149–1.316)	< 0.0001	1.204 (1.119–1.295)	< 0.0001
Categorical eGFRcys/eGFRcr						
≥ 0.6	1.0 (ref)		1.0 (ref)		1.0 (ref)	
< 0.6	2.663 (2.108–3.291)	< 0.0001	1.684 (1.344–2.109)	< 0.0001	1.491 (1.170–1.901)	0.0013
Per 10% decrease	1.169 (1.129–1.212)	< 0.0001	1.086 (1.054–1.120)	< 0.0001	1.072 (1.036–1.108)	< 0.0001
*1‐year time‐updated measures*						
Categorical ΔeGFRdiffcys‐cr, mL/min/1.73 m2						
Tertile 1 (negative change)	1.339 (0.984–1.822)	0.0635	1.500 (1.100–2.045)	0.0103	1.442 (1.039–2.001)	0.0287
Tertile 2 (unchanged)	1.0 (ref)		1.0 (ref)		1.0 (ref)	
Tertile 3 (positive change)	1.296 (0.950–1.768)	0.1016	1.247 (0.914–1.702)	0.1632	1.186 (0.856–1.644)	0.3051

*Note:* Model 1: Analysis adjusted for age, sex, and baseline creatinine‐based eGFR. Model 2: Model 1 plus hypertension, coronary artery disease, diabetes mellitus, hyperlipidemia, smoking, alcohol consumption, body mass index, HDL‐C, LDL‐C, triglyceride, baseline NIHSS score, ACEI/ARB, dehydrant and contrast agent use, and baseline log UACR (data need log transformed).

## 4 Discussion

This study explored the association between the difference in eGFRcys and eGFRcr and stroke prognosis. One‐third of participants had a baseline difference lower than −15 mL/min/1.73 m^2^ between their eGFRcys and eGFRcr values. We observed that a negative baseline eGFRdiffcys‐cr value is significantly associated with a higher risk of post‐stroke disability and all‐cause mortality. Additionally, time‐updated measurements of eGFRdiffcys‐cr provided prognostic information; participants who experienced a faster decline in eGFRcys than eGFRcr had a 1.4 times higher risk of mortality than those with relatively stable ΔeGFRdiffcys‐cr. Collectively, our study results indicate that the difference between eGFRcys and eGFRcr can be used to assess the risk of individual post‐stroke disability and death and stratify and precisely manage the risk of stroke patients. Furthermore, time‐updated results indicate that compared with a one‐time assessment of serum creatinine and cystatin C, repeated measurements can provide more information about changes in self‐rated health status, as the current guideline‐recommended practice [19].

In this analysis, the baseline eGFRabdiff was −9.2 ± 15.5 mL/min/1.73 m^2^, with 35.2% of cases showing a difference lower than −15 mL/min/1.73 m^2^. Consistent with our results, a diabetes cohort (*n* = 25,825) had an eGFRdiff of −11.1 ± 14.1 mL/min/1.73 m^2^, with 38.9% of patients having an eGFRdiff exceeding −15 mL/min/1.73 m^2^ [5]. Additionally, in a healthcare utilization cohort from the Stockholm region, Sweden (*n* = 158,601), the average eGFRdiff was −7 ± 19 mL/min/1.73 m^2^, with 32% of measurements showing a negative eGFRdiff lower than −15 mL/min/1.73 m^2^ [12]. The baseline eGFRcr values in these studies ranged from 80 to 95.6 mL/min/1.73 m^2^. However, these results differed from other studies: In the Chronic Renal Insufficiency Cohort Study (*n* = 4,956), the average eGFRdiff was +6 ± 16 mL/min/1.73 m^2^ [3]; in the Chronic Renal Insufficiency Cohort Study (*n* = 9,092), the average eGFRdiff was −0.5 ± 15 mL/min/1.73 m^2^ [20]; in the Cardiovascular Health Study (*n* = 4,635), the average eGFRdiff was −1.4 ± 14 mL/min/1.73 m^2^ [21]. The ratio of eGFRdiff exceeding −15 mL/min/1.73 m^2^ in these studies ranged from 8% to 16%, with baseline eGFRcr values ranging from 49 to 73 mL/min/1.73 m^2^. Studies with higher baseline eGFRcr often reported larger negative eGFRdiff and a higher proportion of eGFRdiff exceeding −15 mL/min/1.73 m^2^. Therefore, monitoring eGFRdiff remains crucial even with higher baseline eGFRcr.

Up to now, studies on different populations have shown a significant association between eGFRdiff and renal outcomes. For instance, in the Atherosclerosis Risk in Communities Study, large negative eGFRdiff is significantly associated with the risk of end‐stage renal disease, as well as the risk of acute kidney injury in the Stockholm Creatinine Measurements Project [12, 18]. Similarly, in heart failure patients, the larger the negative value of eGFRdiff, the higher the risk of renal function deterioration [13]. In addition, eGFRdiff is closely associated with other adverse prognoses. In the Cardiovascular Health Study cohorts, the negative baseline eGFRdiff was associated with a higher risk of frailty and all‐cause mortality [21]. The Systolic Blood Pressure Intervention Trial focuses on the major cardiovascular adverse events, showing that a lower baseline eGFRdiff was associated with a higher risk of non‐fatal acute myocardial infarction, unstable angina, stroke, congestive heart failure, symptomatic arrhythmias, and cardiogenic death [20]. Data from the CHARLS (China Health and Retirement Longitudinal Study) declared that a large negative eGFRdiff was independently associated with higher prevalence and incidence rates of stroke [11]. Recent studies involving diabetic patients from the UK Biobank showed that baseline eGFRdiff was negatively correlated with the risk of microvascular multimorbidity and macrovascular multimorbidity [5, 6].

There are currently no published studies on the relationship between eGFRdiff and stroke prognosis. Our study showed that the significant differences between eGFRcys and eGFRcr, as well as the widening of these differences over time, were independently associated with an increased risk of post‐stroke disability and mortality. The mechanisms underpinning remain inadequately understood. A study on Health, Aging, and Body Composition study (*n* = 2,970) showed that a lower eGFRdiff is closely associated with reduced muscle mass, poor muscle strength, and impaired functional status [22, 23, 24]. In patients with compromised health status, eGFRcr may show a stable or even increasing trend over time, while eGFRcys is not influenced by such factors. In our study, participants showing a significant negative eGFRdiff had lower triglyceride levels and a lower proportion of individuals with hyperlipidaemia, indicating a possible state of malnutrition. Additionally, stroke‐related sarcopenia, manifested as decreased muscle mass and strength, as well as deteriorated physical fitness and nutritional status, may also lead to falsely stable eGFRcr [25]. In summary, reduced physical activity, sarcopenia, and nutritional deterioration due to stroke may decrease creatinine production, thereby leading to an increase in the eGFRdiff and poor outcomes for stroke [26, 27, 28, 29].

Furthermore, the hypothesis of shrunken pore syndrome (SPS) also provides an additional mechanism for the relationship between the increased negative eGFRdiff and poor prognosis; pathophysiological states of eGFRcys/eGFRcr < 0.6 or < 0.7 suggest selective glomerular filtration injury with cystatin C and other molecules (5–30 kDa) [30, 31]. Proteomic studies on SPS suggest that the high mortality rate of this syndrome is due to the accumulation of 10–30 kDa signaling proteins, which promote the development of atherosclerosis and are associated with adverse outcomes [32, 33]. Therefore, as overall health status deteriorates, the negative eGFRdiff is likely to increase gradually, which may be a possible reason for the association between widening in eGFRdiff and poor stroke prognosis. Notably, the American Society of Nephrology and the National Kidney Foundation strongly recommend routine screening for cystatin C [34], and our study results further emphasize the necessity for continuous measurements of eGFRcys and eGFRcr rather than single measurements of both.

This study is the first to investigate the association between eGFRdiff and stroke outcomes. Our findings suggest that stroke patients often have a larger negative eGFRdiffcys‐cr; relying solely on eGFRcr may not fully capture the risk of adverse stroke outcomes. Additionally, the results of time‐updated eGFRdiffcys‐cr suggest that annual repeated measurements of cystatin C can supply the prognostic risk assessment for stroke patients. By focusing on the interplay between renal function markers and neurological outcomes, our work bridges nephrology and neurology, providing actionable insights for clinicians managing complex stroke populations.

The strengths of this study lie in its nationwide cohort, large sample size, prospective design, 5‐year follow‐up, and repeated assessments of eGFRdiffcys‐cr. Additionally, all blood samples were tested in the same central laboratory, ensuring accurate serum creatinine and cystatin C measurement. However, this study has some limitations. First, we did not directly assess muscle mass, which can independently affect creatinine levels. Additionally, although previous studies have suggested a possible link between cystatin C levels and thyroid dysfunction and inflammation, we lack corresponding data [35, 36]. Second, despite adjusting for potential confounders, we cannot completely rule out the presence of residual confounding factors, such as inflammation or oxidative stress markers not included in the analysis. Third, the study population mostly retained kidney function upon admission, with 96.5% of patients having eGFRcr > 60 mL/min/1.73 m^2^. Therefore, the applicability of these results to late‐stage kidney disease patients in stroke populations requires further research. Fourth, our study participants were all from China; therefore, caution should be exercised when applying our study results to other populations. Fifth, the time‐updated analysis of ΔeGFRdiff was restricted to 4508 participants, accounting for 44% of the baseline cohort; potential selection bias cannot be entirely excluded. Finally, the single baseline measurements of serum creatinine and cystatin C in our study may lead to potential misclassification bias.

## 5 Conclusion

Our study demonstrates that over one‐third of the stroke population has a GFRcys below GFRcr of 15 mL/min/1.73 m^2^ or more. This difference and the widening eGFRdiff over time are closely associated with poor stroke outcomes. Our study results support the regular and separate measurements of eGFRcys and eGFRcr in the long‐term management of stroke patients.

## Author Contributions

Qi Huang and Yilun Zhou were responsible for conception and design of the study, and drafting the article; Yuesong Pan and Hongyi Yan analyzed and interpreted data; Xia Meng, Yin Zhang, Mengxing Wang, and Anxin Wang provided intellectual content of critical importance to the work described; Yilun Zhou and Yongjun Wang are both corresponding authors who provided final approval of the version to be published. The author(s) read and approved the final manuscript.

## Funding

This work was supported by Capital's Funds for Health Improvement and Research (2020‐1‐2041), the Chinese Academy of Medical Sciences Innovation Fund for Medical Sciences (2019‐I2M‐5‐029), the National Natural Science Foundation of China (81870905, U20A20358), and the Beijing Municipal Administration of Hospitals Incubating Program (PX2020021).

## Disclosure

The authors have no conflicts of interest to declare.

## Ethics Statement

The CNSR‐III study was performed in accordance with the Declaration of Helsinki, and was approved by the ethics committee at Beijing Tiantan Hospital (IRB approval no. KY2015‐001‐01) and all participating centers. Written informed consent was obtained from the patient or legally authorized representative (primarily spouse, parents, adult children, otherwise indicated).

## Conflicts of Interest

The authors declare no conflicts of interest.

## Supporting information


**Table S1:** Association of baseline and 1‐year time‐updated eGFRdiffcys‐cr with Stroke recurrence.

## Data Availability

The data that support the findings of this study are openly available in Third China National Stroke Registry at http://creativecommons.org/licenses/by‐nc/4.0/.

## References

[1] M. G. Shlipak , K. Matsushita , J. Ärnlöv , et al., “Cystatin C Versus Creatinine in Determining Risk Based on Kidney Function,” New England Journal of Medicine 69 (2013): 932–943. 10.1056/NEJMoa1214234 PMC3993094 24004120

[2] J. S. Lees , C. E. Welsh , C. A. Celis‐Morales , et al., “Glomerular Filtration Rate by Differing Measures, Albuminuria and Prediction of Cardiovascular Disease, Mortality and End‐Stage Kidney Disease,” Nature Medicine 5 (2019): 1753–1760. 10.1038/s41591-019-0627-8 PMC6858876 31700174

[3] D. C. Chen , M. G. Shlipak , R. Scherzer , et al., “Association of Intraindividual Difference in Estimated Glomerular Filtration Rate by Creatinine vs Cystatin C and End‐Stage Kidney Disease and Mortality,” JAMA Network Open 5 (2022): e2148940, 10.1001/jamanetworkopen.2021.48940. 35175342 PMC8855239

[4] C. Li , Y. Ma , C. Yang , R. Hua , W. Xie , and L. Zhang , “Association of Cystatin C Kidney Function Measures With Long‐Term Deficit‐Accumulation Frailty Trajectories and Physical Function Decline,” JAMA Network Open 5 (2022): e2234208, 10.1001/jamanetworkopen.2022.34208. 36178684 PMC9526088

[5] D. He , B. Gao , J. Wang , C. Yang , M. H. Zhao , and L. Zhang , “The Difference Between Cystatin C‐ and Creatinine‐Based Estimated Glomerular Filtration Rate and Risk of Diabetic Microvascular Complications Among Adults With Diabetes: A Population‐Based Cohort Study,” Diabetes Care 47 (2024): 873–880. 38470988 10.2337/dc23-2364 PMC11043223

[6] F. Chen , Y. Zhang , D. Gao , et al., “Difference Between Cystatin C‐ and Creatinine‐Based Estimated Glomerular Filtration Rate and Risk of Diabetes‐Related Multimorbidity Among Adults With Diabetes,” Diabetes Research and Clinical Practice 227 (2025): 112419, 10.1016/j.diabres.2025.112419. 40812429

[7] H. Kim , J. T. Park , J. Lee , et al., “The Difference Between Cystatin Cand Creatinine‐Based eGFR Is Associated With Adverse Cardiovascular Outcome in Patients With Chronic Kidney Disease,” Atherosclerosis 335 (2021): 53–61. 34571286 10.1016/j.atherosclerosis.2021.08.036

[8] D. He , C. Li , C. Yang , et al., “Difference Between Cystatin C‐ and Creatinine‐Based Estimated Glomerular Filtration Rate, Diabetes Status, and Incident Peripheral Artery Disease: A Population‐Based Cohort Study,” Atherosclerosis 408 (2025): 120452, 10.1016/j.atherosclerosis.2025.120452. 40712416

[9] D. C. Chen , M. G. Shlipak , R. Scherzer , et al., “Association of Intra‐Individual Differences in Estimated GFR by Creatinine Versus Cystatin C With Incident Heart Failure,” American Journal of Kidney Diseases 80 (2022): 762–772. 35817274 10.1053/j.ajkd.2022.05.011 PMC9691565

[10] M. C. IE , J. Yang , A. S. Go , et al., “Complex Etiologies of the Discordance Between Cystatin C‐ and Creatinine‐Based Estimated GFR and Its Adverse Associations: Findings From the CRIC Study,” American Journal of Kidney Diseases 86 (2025): 192–201. 40373998 10.1053/j.ajkd.2025.03.018 PMC12818706

[11] X. Shi , J. Song , F. Chen , et al., “Association of Differences in Cystatin C‐ and Creatinine‐Based Estimated Glomerular Filtration Rate With Prevalence and Incidence of Stroke,” Journal of the American Heart Association 14 (2025): e039185, 10.1161/JAHA.124.039185. 40407072 PMC12229175

[12] J. J. Carrero , E. L. Fu , Y. Sang , et al., “Discordances Between Creatinine‐ and Cystatin C‐Based Estimated GFR and Adverse Clinical Outcomes in Routine Clinical Practice,” American Journal of Kidney Diseases 82 (2023): 534–542. 37354936 10.1053/j.ajkd.2023.04.002

[13] A. Pinsino , M. R. Carey , S. Husain , et al., “The Difference Between Cystatin C‐ and Creatinine Based Estimated GFR in Heart Failure With Reduced Ejection Fraction: Insights From PARADIGM‐HF,” American Journal of Kidney Diseases 82 (2023): 521–533. 37086965 10.1053/j.ajkd.2023.03.005

[14] M. C. IE , A. S. Go , J. Y. Hsu , et al., “Differential Effect of Hospitalization on Cystatin C‐ and Creatinine‐Based Estimated GFR,” Journal of the American Society of Nephrology 36 (2025): 1585–1591. 40067355 10.1681/ASN.0000000670 PMC12342081

[15] D. Kervella , S. Lemoine , F. Sens , et al., “Cystatin C Versus Creatinine for GFR Estimation in CKD due to Heart Failure,” American Journal of Kidney Diseases 69 (2017): 321–323. 27876172 10.1053/j.ajkd.2016.09.016

[16] A. Torre , J. M. Aguirre‐Valadez , J. M. Arreola‐Guerra , et al., “Creatinine Versus Cystatin C for Estimating GFR in Patients With Liver Cirrhosis,” American Journal of Kidney Diseases 67 (2016): 342–344. 10.1053/j.ajkd.2015.09.022 26530874

[17] Y. Wang , J. Jing , X. Meng , et al., “The Third China National Stroke Registry (CNSR‐III) for Patients With Acute Ischaemic Stroke or Transient Ischaemic Attack: Design, Rationale and Baseline Patient Characteristics,” Stroke Vasc Neurol 4 (2019): 158–164. 31709123 10.1136/svn-2019-000242 PMC6812638

[18] D. K. Farrington , A. Surapaneni , K. Matsushita , J. C. Seegmiller , J. Coresh , and M. E. Grams , “Discrepancies Between Cystatin C–Based and Creatinine‐Based Estimated Glomerular Filtration Rates,” Clinical Journal of the American Society of Nephrology 18 (2023): 1143–1152. 37339177 10.2215/CJN.0000000000000217 PMC10564370

[19] P. E. Stevens and A. Levin , “Evaluation and Management of Chronic Kidney Disease: Synopsis of the Kidney Disease: Improving Global Outcomes 2012 Clinical Practice Guideline,” Annals of Internal Medicine 158 (2013): 825–830. 23732715 10.7326/0003-4819-158-11-201306040-00007

[20] O. A. Potok , J. H. Ix , M. G. Shlipak , et al., “The Difference Between Cystatin C‐ and Creatinine Based Estimated GFR and Associations With Frailty and Adverse Outcomes: A Cohort Analysis of the Systolic Blood Pressure Intervention Trial (SPRINT),” American Journal of Kidney Diseases 76 (2020): 765–774. 32682697 10.1053/j.ajkd.2020.05.017 PMC8896529

[21] O. A. Potok , R. Katz , N. Bansal , et al., “The Difference Between Cystatin C‐ and Creatinine‐Based Estimated GFR and Incident Frailty: An Analysis of the Cardiovascular Health Study (CHS),” American Journal of Kidney Diseases 76 (2020): 896–898. 32682698 10.1053/j.ajkd.2020.05.018 PMC7967899

[22] D. H. Seo , Y. J. Suh , Y. Cho , et al., “Effect of Low Skeletal Muscle Mass and Sarcopenic Obesity on Chronic Kidney Disease in Patients With Type 2 Diabetes,” Obesity (Silver Spring) 30 (2022): 2034–2043. 36062861 10.1002/oby.23512

[23] D. Purnamasari , E. N. Tetrasiwi , G. J. Kartiko , C. Astrella , K. Husam , and P. W. Laksmi , “Sarcopenia and Chronic Complications of Type 2 Diabetes Mellitus,” Review of Diabetic Studies 18 (2022): 157–165. 10.1900/RDS.2022.18.157 PMC9652710 36309772

[24] O. A. Potok , J. H. Ix , M. G. Shlipak , et al., “Cystatin C‐ and Creatinine‐Based Glomerular Filtration Rate Estimation Differences and Muscle Quantity and Functional Status in Older Adults: The Health, Aging, and Body Composition Study,” Kidney Medicine 4 (2022): 100416, 10.1016/j.xkme.2022.100416. 35386603 PMC8978136

[25] C. Y. Huang and Y. H. Liu , “Sex Difference, Proteostasis and Mitochondrial Function Impact Stroke‐Related Sarcopenia‐A Systematic Review and Meta‐Analysis,” Ageing Research Reviews 101 (2024): 102484, 10.1016/j.arr.2024.102484. 39218079

[26] K. E. Ensrud , N. Parimi , H. A. Fink , et al., “Estimated GFR and Risk of Hip Fracture in Older Men: Comparison of Associations Using Cystatin C and Creatinine,” American Journal of Kidney Diseases 63 (2014): 31–39. 23890927 10.1053/j.ajkd.2013.05.022 PMC3833961

[27] L. S. Dalrymple , R. Katz , D. E. Rifkin , et al., “Kidney Function and Prevalent and Incident Frailty,” Clinical Journal of the American Society of Nephrology 8 (2013): 2091–2099. 24178972 10.2215/CJN.02870313 PMC3848393

[28] S. Beddhu , M. H. Samore , M. S. Roberts , G. J. Stoddard , L. M. Pappas , and A. K. Cheung , “Creatinine Production, Nutrition, and Glomerular Filtration Rate Estimation,” Journal of the American Society of Nephrology 14 (2003): 1000–1005. 12660334 10.1097/01.asn.0000057856.88335.dd

[29] S. H. Ballew , Y. Chen , N. R. Daya , et al., “Frailty, Kidney Function, and Polypharmacy: The Atherosclerosis Risk in Communities (ARIC) Study,” American Journal of Kidney Diseases 69 (2017): 228–236. 27884475 10.1053/j.ajkd.2016.08.034 PMC5263025

[30] A. Grubb , “Shrunken Pore Syndrome ‐ a Common Kidney Disorder With High Mortality. Diagnosis, Prevalence, Pathophysiology and Treatment Options,” Clinical Biochemistry 83 (2020): 12–20. 32544475 10.1016/j.clinbiochem.2020.06.002

[31] L. Malmgren , C. Öberg , E. den Bakker , et al., “The Complexity of Kidney Disease and Diagnosing It ‐ Cystatin C, Selective Glomerular Hypofiltration Syndromes and Proteome Regulation,” Journal of Internal Medicine 293 (2023): 293–308. 36385445 10.1111/joim.13589 PMC10107454

[32] T. Y. Wong , C. M. Cheung , M. Larsen , S. Sharma , and R. Simó , “Diabetic Retinopathy,” Nature Reviews. Disease Primers 2 (2016): 16013 Nature Reviews Disease Primers. 10.1038/nrdp.2016.12 27159554

[33] K. R. Tuttle , R. Agarwal , C. E. Alpers , et al., “Molecular Mechanisms and Therapeutic Targets for Diabetic Kidney Disease,” Kidney International 102 (2022): 248–260. 35661785 10.1016/j.kint.2022.05.012

[34] C. Delgado , M. Baweja , D. C. Crews , et al., “A Unifying Approach for GFR Estimation: Recommendations of the NKF‐ASN Task Force o Reassessing the Inclusion of Race in Diagnosing Kidney Disease,” Journal of the American Society of Nephrology 32 (2021): 2994–3015. 34556489 10.1681/ASN.2021070988 PMC8638402

[35] M. Cañadas‐Garre , K. Anderson , J. McGoldrick , A. P. Maxwell , and A. J. McKnight , “Genomic Approaches in the Search for Molecular Biomarkers in Chronic Kidney Disease,” Journal of Translational Medicine 16 (2018): 292. 30359254 10.1186/s12967-018-1664-7 PMC6203198

[36] E. Iversen , A. C. Bodilsen , H. H. Klausen , et al., “Kidney Function Estimates Using Cystatin C Versus Creatinine: Impact on Medication Prescribing in Acutely Hospitalized Elderly Patients,” Basic & Clinical Pharmacology & Toxicology 124 (2019): 466–478. 30372593 10.1111/bcpt.13156

