# Supplementary material for: Association Between Differences in Estimated GFR by Creatinine Vs. Cystatin C and Stroke Outcomes: Results From the Third China National Stroke Registry
Source: CNS Neurosci Ther. 2026 May 18;32(5):e70930. doi: 10.1002/cns.70930 (PMC13182591; doi:10.1002/cns.70930)
Supplement: Supplementary file 1 — Table S1: Association of baseline and 1‐year time‐updated eGFRdiffcys‐cr with Stroke recurrence. [file CNS-32-e70930-s001.docx]

Supplemental Table S1. Association of baseline and 1 year time-updated eGFRdiffcys-cr with Stroke recurrence

| Measure | Unadjusted | | Model 1 | | Model 2 | |
| --- | --- | --- | --- | --- | --- | --- |
|  | OR/HR  (95%CI) | *p* value | OR/HR  (95%CI) | *p* value | OR/HR  (95%CI) | *p* value |
| **Baseline measures** |  |  |  |  |  |  |
| **Categorical eGFRdiffcys-cr, mL/min/1.73 m2** |  |  |  |  |  |  |
| ＜−15 | 1.030(0.930-1.141) | 0.57043 | 0.991(0.893-1.099) | 0.8582 | 0.993(0.890-1.109) | 0.9066 |
| -15 to 15 | 1.0(ref) |  | 1.0(ref) |  | 1.0(ref) |  |
| ≥ 15 | 1.020(0.816-1.274) | 0.8629 | 0.982(0.779-1.239) | 0.8807 | 1.034(0.800-1.336) | 0.7993 |
| Per 1-SD decrease | 0.982 (0.936-1.031) | 0.4659 | 1.000(0.996-1.003) | 0.8097 | 1.000(0.996-1.003) | 0.8224 |
| **Categorical eGFRcys / eGFRcr** |  |  |  |  |  |  |
| ≥0.6 | 1.0(ref) |  | 1.0(ref) |  | 1.0(ref) |  |
| ＜0.6 | 1.119(0.865-1.448) | 0.3919 | 0.999(0.771-1.295) | 0.9930 | 1.019(0.780-1.332) | 0.8904 |
| Per 10% decrease | 0.988 (0.966-1.011) | 0.4659 | 0.996 (0.974-1.019) | 0.7187 | 1.000(0.974-1.027) | 0.9761 |
| **1 year time-updated measures** |  |  |  |  |  |  |
| **Categorical ΔeGFRdiffcys-cr, mL/min/1.73 m2** |  |  |  |  |  |  |
| Tertile 1 (negative change) | 1.220(1.020-1.459) | 0.0298 | 1.245(1.039-1.491) | 0.0177 | 1.160(0.957-1.406) | 0.1304 |
| Tertile 2 (unchanged) | 1.0(ref) |  | 1.0(ref) |  | 1.0(ref) |  |
| Tertile 3 (positive change) | 0.973(0.806-1.174) | 0.7718 | 0.965(0.800-1.165) | 0.7131 | 0.948(0.779-1.155) | 0.5984 |

**Model 1**: Analysis adjusted for age, sex, and baseline creatinine-based eGFR.

**Model 2**: Model 1 plus hypertension, coronary artery disease, diabetes mellitus, hyperlipidemia, smoking, alcohol consumption, body mass index, HDL-C, LDL-C, triglyceride, baseline NIHSS score, ACEI/ARB, dehydrant and contrast agent use and baseline log UACR (data need log transformed).
